# An *in vivo* fluorescence imaging approach for real-time monitoring of ROS-associated oxidative responses in harvested lettuce leaves

**DOI:** 10.3389/fpls.2026.1923387

**Published:** 2026-10-19

**Authors:** Mohamed Hawali Bata Gouda, Christophe B. Y. Cordella, Arturo Duarte-Sierra

**Affiliations:** 1 Food Science Department, Laval University, Quebec, QC, Canada; 2 Centre de Recherche en Science du Végétal (Centre SÈVE), Sherbrooke University, Sherbrooke, QC, Canada; 3 Institute of Nutrition and Functional Foods (INAF), Laval University, Quebec, QC, Canada; 4 Center for Research in Plant Innovation (CRIV), Laval University, Quebec, QC, Canada; 5 Laboratoire de Recherche et de Traitement de l'Information Chimiosensorielle (LARTIC), Laval University, Quebec, QC, Canada

**Keywords:** fluorescence, *in vivo* imaging, oxidative stress, postharvest, probes, ROS

## Abstract

Reactive oxygen species (ROS) play a key role in postharvest quality and senescence of fresh produce, yet their real-time monitoring in intact tissues remains challenging. In this study, we developed and evaluated an *in vivo* fluorescence imaging approach for monitoring ROS dynamics in whole harvested lettuce leaves using the ROS-sensitive probe 2’, 7’-dichlorodihydrofluorescein diacetate (H_2_DCF-DA). The stability of the probe and the sensitivity of the imaging system were first assessed under a tissue-free condition. Subsequently, the method was applied to whole lettuce leaves subjected to exogenous hydrogen peroxide (H_2_O_2_) or ultraviolet-C (UV-C) irradiations at doses of 0.85, 1.71 and 3.42 kj/m^2^. A strong and stable fluorescence signal in the presence of H_2_O_2_ under tissue-free conditions was observed, confirming probe reactivity and the system detection sensitivity. Exogenous H_2_O_2_ application in whole lettuce leaves, resulted in a progressive increase in ROS-associated fluorescence, whereas UV-C irradiation induced a rapid and dose-dependent fluorescence response followed by a gradual decline, indicative of a transient oxidative burst and subsequent redox regulation. These findings demonstrate that *in vivo* fluorescence imaging is a sensitive and non-invasive tool for real-time monitoring of ROS in harvested produce. This approach offers new perspectives for investigating oxidative stress dynamics relevant to postharvest quality management of fruits and vegetables.

## 1 Introduction

Reactive oxygen species (ROS) are a chemically diverse group of oxygen-derived molecules capable of inducing a wide range of cellular modifications. They include both radical species such as superoxide anion (O₂•⁻), hydroxyl radical (HO•), peroxyl radical (ROO•) and alkoxyl radical (RO•) and non-radical species such as hydrogen peroxide (H_2_O_2_), and singlet oxygen (^1^O_2_) (Choudhury et al., 2017; Gill and Tuteja, 2010; Muñoz and Munné-Bosch, 2018). In plant tissues, ROS are continuously generated as by-products of aerobic metabolism in several cellular compartments. Major production sites include the mitochondria where the electron transport chain leaks electrons to molecular oxygen; the chloroplasts particularly at the photosystem I and II during photosynthetic electron transport; and the peroxisomes through photorespiration and oxidase reactions (Gómez et al., 2019). They differ in reactivity, stability, diffusion capacity, and signaling potential (Muñoz and Munné-Bosch, 2018; Rubio and Cerón, 2021). At the molecular level, ROS exert a dual function serving as signaling molecules at controlled concentrations and as potent oxidizing agents that can damage lipids, proteins and nucleic acids when present in excess (Miller et al., 2010; Suzuki and Mittler, 2006). In fresh produce, this redox imbalance accelerates senescence and contributes to postharvest quality deterioration (Tian et al., 2013; Wang et al., 2019). Therefore, monitoring ROS dynamics is essential for understanding oxidative stress mechanisms, redox signaling pathways, and their contribution to postharvest senescence and quality deterioration.

Numerous analytical methods have been developed for ROS detection and quantification, ranging from conventional methods such as spectrophotometry (Tan et al., 2025) and chromatography (Egwu et al., 2021), to electrochemical methods (Huo et al., 2022), fluorescence and chemiluminescence-based approaches (Pring et al., 2023; Wu et al., 2019). Although these approaches have significantly contributed to the understanding of oxidative processes in biological systems, many still require destructive sample preparation and rely on endpoint measurements, limiting their ability to capture spatial and temporal dynamics of ROS in living tissues (Bata Gouda et al., 2025; Zhang et al., 2018). In contrast, fluorescence-based methods offer high sensitivity and enable real-time monitoring of ROS using fluorescent probes. These probes differ in their specificity and reactivity toward ROS. In many cases, they remain non-fluorescent until undergoing ROS-mediated oxidation, leading to the generation of a detectable fluorescence signal (Maeda et al., 2004; Soh, 2006; Winterbourn, 2013). Depending on the probe, the resulting fluorescence may reflect either overall oxidative activity or the presence of specific ROS. For instance, 2’, 7’-Dichlorofluorescin and its diacetyl derivative (H_2_DCF-DA) are widely used as general indicators of intracellular ROS, whereas Amplex Red is more specific for the detection of H_2_O_2_. In addition, dihydroethidium (DHE) and its mitochondria-targeted derivative MitoSOX are commonly employed to monitor O_2_^·^ production. However, fluorescence-based methods are subject to probe-specific limitations. Notably, H_2_DCF-DA, despite its high sensitivity and ease of use, is susceptible to auto-oxidation and photo-oxidation, which may lead to overestimation of the resulting fluorescence signal.

Recent advances in optical imaging have further expanded the capabilities of fluorescence-based methods by enabling their integration with imaging techniques such as confocal laser scanning microscopy (CLSM) and fluorescence lifetime imaging microscopy (FLIM), thereby facilitating the spatial and temporal visualization of ROS dynamics in living tissues (Khaliduzzaman et al., 2023; Takbhate, 2024). However, these approaches remain limited to relatively small tissue regions, restricting the investigation of oxidative processes at the whole-organ level (Icha et al., 2017). Such limited spatial coverage constrains the characterization of the overall oxidative status of the intact plant tissues and hampers a comprehensive understanding of coordinated oxidative stress responses at the organismal level.

To overcome these limitations, highly sensitive *in vivo* optical imaging platforms have been designed and have demonstrated their ability to monitor ROS non-invasively at the whole-plant scale (Fichman et al., 2019). These platforms provide real-time, spatially resolved information on oxidative processes within intact plants. Such systems typically incorporate an ultrasensitive camera, either a charge-coupled device (CCD) or an electron-multiplying charge-coupled device (EMCCD) combined with excitation/emission filters, allowing for operation across a wide spectral range with high contrast and temporal resolution (Bata Gouda et al., 2026; Brovko, 2010). They enable bioluminescence and fluorescence detection as well as ultraweak photon emission (UPE) and delayed luminescence (DL). Among these imaging modalities, fluorescence imaging has emerged as a particularly powerful approach for the non-destructive monitoring of ROS using sensitive fluorogenic probes.

While this approach has demonstrated strong potential for visualizing ROS dynamics in whole plants, its application in postharvest research remains largely limited. Therefore, this study aimed to develop and evaluate an *in vivo* fluorescence imaging workflow for the non-destructive and time-resolved monitoring of ROS-associated oxidative responses in postharvest produce. Experimental conditions were first optimized to ensure reliable fluorescence detection and quantitative analysis of stress-induced ROS responses. The feasibility of the method was then evaluated using oxidative stress treatments to induce ROS production in harvested lettuce leaves. This approach provides a valuable experimental platform for investigating ROS dynamics in postharvest produce and improving the understanding of oxidative stress associated with postharvest storage and quality deterioration.

## 2 Materials and methods

### 2.1 Plant materials

In this study, lettuce leaves were used as model plant material. Whole heads of lettuce (*Lactuca sativa* L. var. *Crispa*) were purchased from a nearby supermarket and transported to the laboratory within 5 min. The leaves from the outer 4 to 6 layers were removed and discarded to reduce bias due to surface contamination and mechanical damage. Only inner leaves devoid of apparent lesions were selected. They were removed from the petiole base, gently washed with distilled water, and stored at 1 ± 0.5 °C for at least 12 hours before use. This storage condition was used as a pre-experimental conditioning step before the experimental procedures. The experiments were carried out by placing the leaf on a non-fluorescent support, adaxial surface facing upward.

### 2.2 Preparation of H_2_DCF-DA stock and working solution

The fluorescent probe H_2_DCF-DA (ThermoFisher Scientific, Invitrogen; catalog no. D399), known to react with multiple types of ROS, was used in this study to enable the detection of a broad range of oxidative signals. A 10 mM stock solution of H_2_DCF-DA was prepared by dissolving 4.87 mg of H_2_DCF-DA in 1 mL of dimethyl sulfoxide (DMSO), aliquoted, and stored at -20 °C until use.

For the preparation of the working solution, 50 µL of the stock solution was diluted in 9950 µL of 50 mM sodium phosphate buffer (pH 7.4), to obtain a final concentration of 50 µM in a total volume of 10 mL. This working concentration was selected based on preliminary optimization experiments in tissue-free and lettuce leaf systems, considering fluorescence signal intensity, temporal stability, signal-to-background ratio (SBR), signal-to-noise ratio (SNR), and fluorescence kinetics (**Supplementary Figure S1**).

### 2.3 Experimental workflow for ROS-associated *in vivo* fluorescence imaging

The *in vivo* fluorescence imaging workflow used in this study was adapted from Fichman et al. (2019), with modifications to accommodate different experimental objectives specific to the postharvest context. Lettuce leaves were fumigated with ROS-responsive fluorescent probes prior to imaging. Depending on the experimental objective, oxidative stress may be applied either before imaging (**Figure 1a**) or after acquisition of a pre-stress fluorescence image (**Figure 1b**). In the first configuration, the first image acquired before fluorescence recording is used as the background reference to correct the total fluorescence, allowing the quantification of total ROS-associated fluorescence. Whereas in the second configuration the pre-stress fluorescence image is used as the background reference for the quantification of stress-associated ROS-dependent fluorescence. Following stress application, fluorescence images are acquired at predefined time intervals and analyzed using the corresponding background correction strategy.

**Figure 1:**
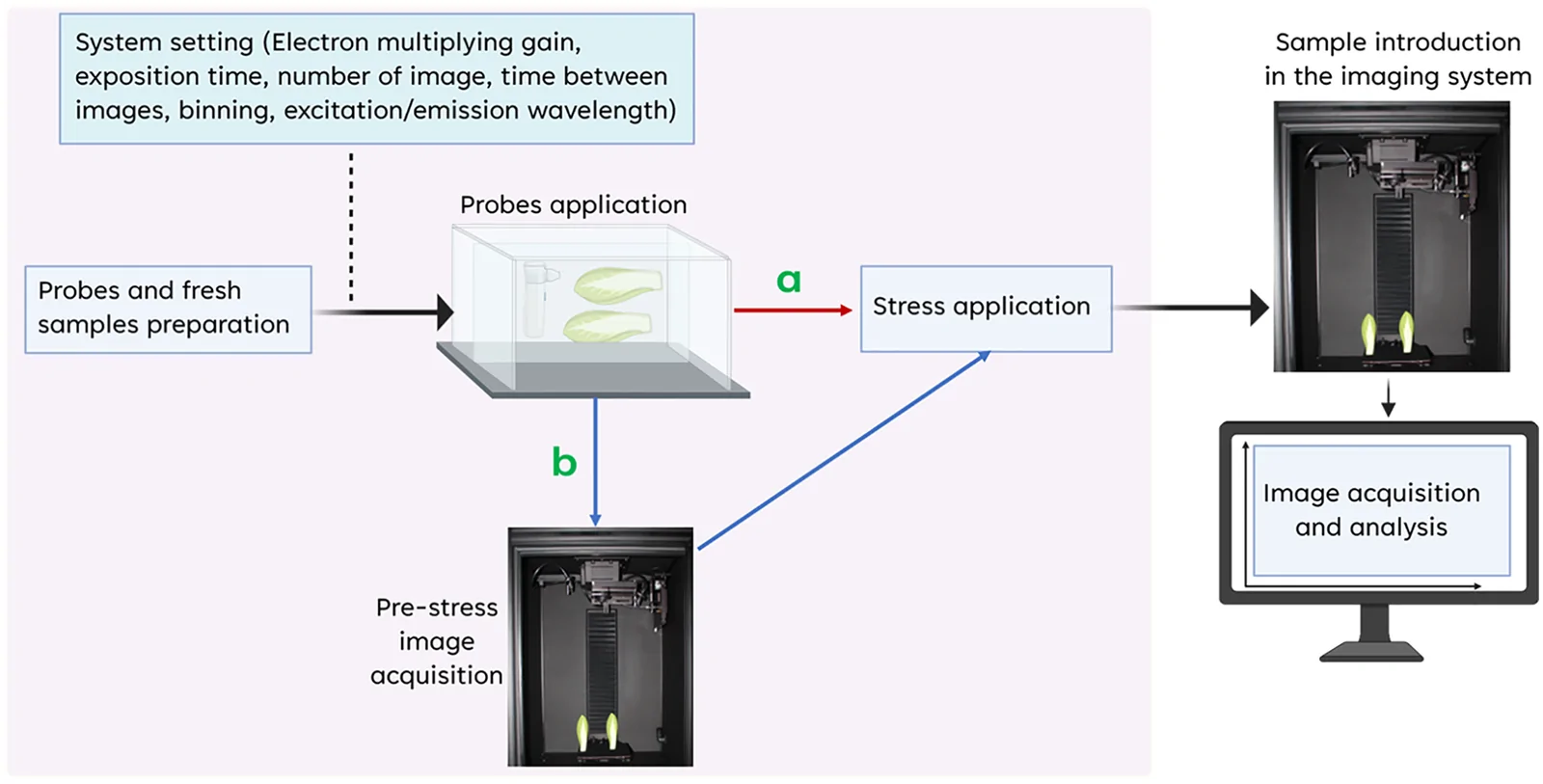
Experimental workflow for *in vivo* fluorescence imaging of ROS-associated oxidative responses in a postharvest context. This workflow illustrates two experimental configurations. Path **(a)** (red arrow) was used to quantify the overall ROS-associated fluorescence in intact tissues, with the first acquired image serving as the background reference for corrected total fluorescence (CTF) calculation. Path **(b)**, (blue arrow) was used to quantify stress-induced changes in ROS-associated fluorescence, with a pre-stress image used as the background reference for CTF calculation.

### 2.4 Experimental validation of the *in vivo* fluorescence imaging approach

To assess the applicability of the *in vivo* fluorescence imaging approach for monitoring ROS dynamics in postharvest systems, two complementary test levels were employed (**Table 1**). First, a tissue-free system was used to assess probe stability, sensitivity, and fluorescence response to controlled ROS generation, thereby validating the fluorescence detection component of the system under controlled conditions. Second, lettuce leaves were used as a case study to evaluate ROS-associated fluorescence response under biologically relevant oxidative stresses, including chemical (H_2_O_2_ application) and physical (UV-C irradiation) treatments.

**Table 1:** Summary of experimental design and tested concentrations.

Test level	Medium	Fluorophore	ROS inducer	Probe concentration (µM)	Exposure time (min)
Probe stability	Well-plate	H_2_DCF-DA	H_2_O_2_	50	120
Case Study	Lettuce leaves	H_2_DCF-DA	H_2_O_2_	50	120
			UV-C		

#### 2.4.1 Probe sensitivity assessment in a tissue-free condition

Probe oxidation was induced by adding 20 µL of 200 µM of H_2_O_2_ to 980 µL of 50 µM H_2_DCF-DA in a well-plate. A control well containing H_2_DCF-DA without hydrogen peroxide was included in parallel.

The experiments were carried out by following the framework described in **Figure 1a**. This assay was used to evaluate signal activation kinetics, detectability, and temporal stability of the signal in the absence of a biological matrix effect.

#### 2.4.2 Induction of oxidative stress in lettuce leaves

Oxidative stress was induced in postharvest lettuce leaves using either a chemical or a physical stressor, following the workflow illustrated in **Figure 1b**. For all stress treatments, lettuce leaves treated with H_2_DCF-DA were first imaged prior to stress application to acquire a baseline fluorescence image, which was used as an internal background reference in subsequent image analysis.

Chemical oxidative stress was induced by external application of H_2_O_2_. The lettuce leaves were uniformly nebulized with 10 mL of 50 µM H_2_DCF-DA. Afterward, 1 mL of 200 µM H_2_O_2_ solution was applied to the leaves using a nebulizer, and fluorescence imaging was resumed to monitor stress-induced ROS production over time.

For physical induction of oxidative stress, leaves were exposed to UV-C irradiation, a highly oxidative stressor. UV-C doses of 0.85, 1.71 and 3.42 kJ/m^2^ doses were selected based on a reported postharvest study on lettuce (Attia et al., 2021). These doses were achieved using a UV-C irradiance of 5.8 mW/cm^2^, with approximate exposure times of 15, 31, and 61 s, respectively. Following UV-C exposure, samples were immediately transferred to the imaging chamber, where fluorescence imaging was performed to monitor ROS production induced by the applied stress.

In all cases, fluorescence signals were quantified after background correction using the pre-stress image, allowing the assessment of stress-associated ROS-dependent fluorescence in postharvest tissues.

### 2.5 Image acquisition

Fluorescence imaging was performed using the PRISM™ *In Vivo* Imaging System (MediLumine Inc., Montreal, Québec, Canada) equipped with an electron-multiplied charge-coupled device (EMCCD) camera and operated in epi-fluorescence mode. H_2_DCF-DA fluorescence was acquired using a 494/25 nm excitation filter and a 525/18 nm emission filter. Images were acquired for 120 min using the epi-fluorescence acquisition preset with an exposure time of 10 s, a binning factor of 1x1 and a constant field of view of 17 cm. Prior to each acquisition, the PRISM software automatically performed a pre-scan to optimize the electron-multiplying gain (EMgain) while minimizing detector saturation. Consequently, the effective EMgain varied among images. The final EMgain value recorded in the image metadata for each acquisition was extracted and incorporated into the radiance calculations (see Equation 2).

Unless otherwise stated, all lettuce experiments were performed using six independent biological replicates. Each biological replicate consisted of a single leaf collected from a different lettuce head and imaged independently under identical imaging conditions. The tissue-free fluorescence assay was performed using three independent experiments.

### 2.6 Image and data analysis

Raw images were processed using a custom Python-based image analysis pipeline (**Supplementary Figure S2**) developed in Python (version 3.12.13) for regions of interest (ROIs) definition, background correction, radiance calculation and quantitative fluorescence analysis. Regions of interest (ROIs) corresponding to individual lettuce leaves were automatically segmented from the reference image and propagated throughout each time-resolved image sequence. Fluorescence intensity was quantified using a background-corrected ROI-based approach adapted from (Gomes et al., 2021). The corrected total fluorescence (CTF) was calculated according to Equation 1:

(1) $$CTF=ID-\left(A_{ROI}\times F_{background}\right)$$

Where:

CTF is the corrected total fluorescence in analog-digital units; ID is the integrated density of the ROI; A_ROI_ is the ROI area and F_background_ is the mean fluorescence intensity of the background.

In this calculation, the background term corresponded to the reference image defined by the experimental configuration (see experimental workflow section) and was used to remove non-specific and pre-existing fluorescence contributions.

To allow comparison among samples, CTF values were converted to radiance units and expressed in photons/s/cm^2^/sr using the relationship reported by Bata Gouda et al. (2026):

(2) $$S_{radiance}=\frac{CTF\times K_{gain}\times d^{2}}{EMgain\times QE\times T\times A_{ROI}\times A_{lens}}$$

Where: CTF represents the corrected fluorescence signal in analog-digital units; K_gain_ is the camera conversion gain; d is the camera-to-sample distance cm; EMgain is the effective electron-multiplying gain obtain from the system; QE is the quantum efficiency of the detector, T is the exposure time in s; A_ROI_ is the area of the ROI in cm^2^ calculated as the product of the pixel in the ROI (ROI_pixel_) and the area of a single pixel (A_pixel_) and A_lens_ is the area of the lens in cm^2^.

Fluorescence intensity is interpreted as a relative indicator of ROS-associated oxidative activity. Time-resolved fluorescence image montages were assembled using ImageJ (Fiji) to visualize spatial and temporal changes in fluorescence intensity.

Time-course fluorescence data were plotted as mean fluorescence intensity of six biological replicates. For statistical comparisons, fluorescence values at selected time points (0, 30, 60, 90 and 120 min) were expressed as mean ± standard deviation (SD) of six biological replicates. For exogenous H_2_O_2_ application experiments, the difference between control and treated samples was assessed using Student's t-test (p < 0.05).

For UV-C experiments, differences among treatments at selected time points were evaluated using one-way analysis of variance (ANOVA) followed by Tukey’s *post hoc* test (p<0.05). The overall fluorescence response throughout the imaging period was evaluated by calculating the area under the fluorescence-time curve (AUC) for each biological replicate using the trapezoidal rule over the 120 min acquisition period. Statistical analyses were also performed in Python (Version 3.12.13) with the NumPy, SciPy, and Statsmodels libraries.

## 3 Results and discussion

In postharvest research, oxidative stress is commonly assessed through the quantification of ROS such as H_2_O_2_ or O_2_·, at specific storage time points using approaches that necessitate sample destruction. While these approaches provide valuable information on the evolution of oxidative status during storage, they may not fully capture early and transient oxidative events that can occur immediately following stress application. In this context, an *in vivo* imaging system, which enables sensitive whole-organ fluorescence imaging, was used in this study to monitor ROS-associated oxidative activity in whole lettuce leaves following stress application.

The proposed workflow enabled the quantification of either the overall ROS-associated fluorescence present in the tissue or oxidative responses specifically induced by an applied stress, depending on the timing of baseline fluorescence acquisition relative to stress exposure (**Figure 1**). The effect of the selected background reference on fluorescence quantification is illustrated in **Supplementary Figure S3**, which shows how different background acquisition times could result in distinct fluorescence dynamics and influence the interpretation of ROS-associated fluorescence dynamics. In addition, when a pre-stress image was used as the background reference, CTF values could become negative if the measured fluorescence following stress application remained below the pre-stress baseline. In this context, negative values do not represent negative photon emission but rather indicate a decrease in ROS-associated fluorescence relative to the initial fluorescence. Depending on the experimental objective, oxidative stress can be induced either before or after fluorophore application to assess cumulative oxidative effects or monitor ROS dynamics. This flexibility allows the workflow to be adapted to a wide range of postharvest studies, including storage, senescence, quality deterioration, and stress responses in different fresh produce.

In our study, the fluorogenic probe H_2_DCF-DA was employed as a general indicator of ROS-associated oxidative processes, with fluorescence arising from probe oxidation by ROS (de Haan et al., 2022). The resulting signal reflects ROS accumulation and allows assessment of stress-induced responses over time without identification of any specific ROS. The experimental approach was used in a tissue-free system to assess probe sensitivity, signal stability, and the capacity of the imaging system to detect the emitted fluorescence under controlled ROS generation using H_2_O_2_ as a controlled source of ROS. It was then applied to intact lettuce leaves to monitor ROS-associated fluorescence following exogenous H_2_O_2_ application and UV-C irradiation, the latter being used to induce endogenous ROS production.

### 3.1 Validation of H_2_DCF-DA fluorescence response in a tissue-free system

Under the tissue-free conditions, the control sample containing H_2_DCF-DA alone exhibited a slight and approximately linear increase in fluorescence, whereas the addition of H_2_O_2_ resulted in a strong and sustained fluorescent signal (**Figures 2A, B**). The limited fluorescence increase observed in the control was likely due to light-induced artifact or auto-oxidation, recognized limitations of DCF-based probes (Kristiansen et al., 2009; Reiniers et al., 2017). Following H_2_O_2_ addition, a clear increase in fluorescence signal was observed over the acquisition period (**Figure 2A**), indicating rapid activation of the probe under oxidizing conditions.

**Figure 2:**
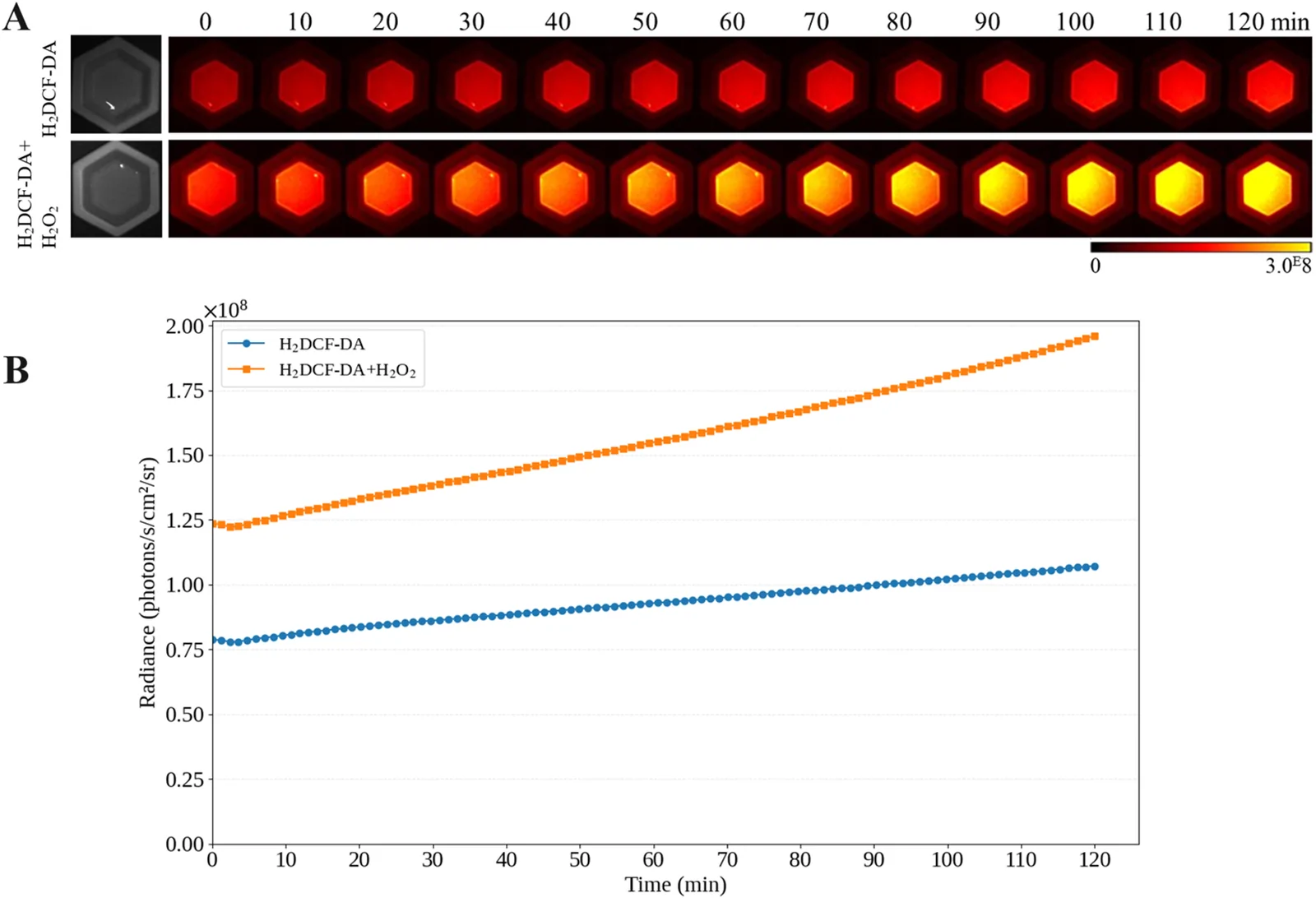
Tissue-free fluorescence response of H_2_DCF-DA at 50 µM. **(A)** Representative fluorescence images from a single biological replicate acquired in the absence (H_2_DCF-DA) and presence of H_2_O_2_ (H_2_DCF-DA+H_2_O_2_). The color scale represents radiance values (photons/sec/cm^2^/sr). **(B)** Time-resolved quantification of fluorescence intensity, expressed as mean radiance (photons/sec/cm^2^/sr) as a function of time using three independent experiments (n = 3).

These results demonstrate that H_2_DCF-DA generates a stable fluorescence response detectable by the imaging system under controlled oxidizing conditions, supporting its use in subsequent postharvest tissue experiments.

### 3.2 ROS-associated fluorescence dynamics in lettuce leaves following exogenous H_2_O_2_ application

In a biological context, an increase in fluorescence intensity was observed in both control and H_2_O_2_-treated samples during the early phase of acquisition (**Figures 3A, B**). This behavior is consistent with the intrinsic physiology of fresh plant tissues, which naturally produce ROS at regulated concentrations as part of normal metabolic activity and redox signaling processes (Mittler, 2017; Sipari et al., 2020). Therefore, a non-zero baseline fluorescence is expected when using ROS-sensitive probes in living systems, and the slight but detectable increase in fluorescence during the early phase in the control can be attributed to the reaction of H_2_DCF-DA with endogenously produced ROS. Notably, this early increase resulted in no significant difference in fluorescence intensity between the control and H_2_O_2_-treated samples (p>0.05), indicating comparable basal ROS levels before the onset of the H_2_O_2_-induced oxidative response (**Figure 3C**). Similar basal fluorescence has been reported in previous DCF-based ROS studies and likely reflects both endogenous ROS production and the known susceptibility of H_2_DCF-DA to non-specific oxidation (Figueroa et al., 2018; Rastogi et al., 2010; Soares et al., 2019; Yazdani, 2015).

**Figure 3:**
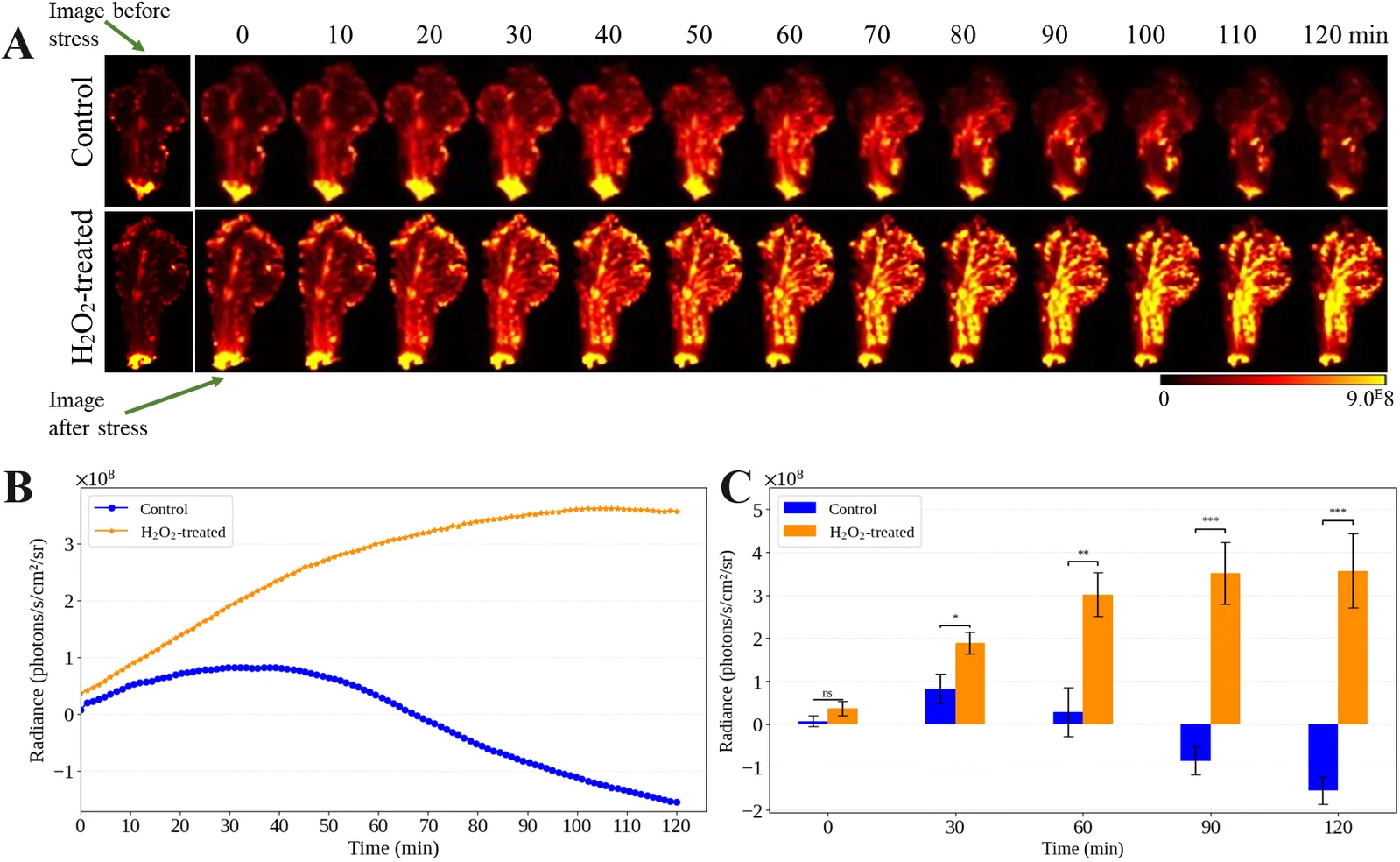
Temporal dynamics of ROS accumulation in lettuce leaves following exogenous H_2_O_2_. **(A)** Representative fluorescence images from a single biological replicate in the absence (control) and presence of H_2_O_2_ (H_2_O_2_-treated). Fluorescence intensity reflects the accumulation of ROS detected by H_2_DCF-DA oxidation. The color scale represents radiance values (photons/sec/cm^2^/sr). **(B)** Time-resolved quantification of fluorescence intensity, expressed as mean radiance (photons/sec/cm^2^/sr) as a function of time using six biological replicates (n = 6). **(C)** Comparison of fluorescence intensity between control and H_2_O_2_-treated lettuce leaves at selected time points (0, 30, 60, 90 and 120 min) based on six biological replicates (n = 6). Statistical differences were evaluated using Student’s t-test. Asterisks indicate significant differences between treatments (***p<0.001, **p<0.01, *p<0.05; ns, not significant).

As shown in **Figures 3A, B**, this pattern was followed by a gradual decline in the control samples, whereas the addition of H_2_O_2_ as an exogenous oxidative source resulted in a higher fluorescence signal that increased rapidly before gradually approaching a plateau after approximately 90 min of acquisition. In addition, our results further indicated that following the initial non-significant fluorescence increase observed in both groups, H_2_O_2_ treatment exhibited significantly higher fluorescence than the control (**Figure 3C**). This observation was further supported by the AUC analysis, which revealed a significantly greater cumulative fluorescence response over the 120-min acquisition period period in H_2_O_2_-treated lettuce leaves than in the control (**Supplementary Figure S4B**). These results indicate that exogenous H_2_O_2_ promoted ROS-associated fluorescence beyond the basal oxidative activity observed in untreated tissues.

The subsequent decrease in signal intensity in the control and the gradual stabilization of the fluorescence signal in H_2_O_2_-treated samples could be interpreted considering the intrinsic capacity of plants to regulate their oxidative status using antioxidant defense systems, including enzymatic (e.g. superoxide dismutase (SOD), catalase (CAT), ascorbate peroxidase (APX)) and non-enzymatic (e.g. Ascorbate (AsA), glutathione, carotenoids) antioxidants (Meitha et al., 2020; You and Chan, 2015). For instance, SOD catalyzes the dismutation of O_2_^·^ into H_2_O_2_, which is subsequently detoxified into H_2_O by CAT or APX (Apel and Hirt, 2004). In contrast, the sustained fluorescence following H_2_O_2_ treatment is likely attributable to the continuous availability of exogenously supplied H_2_O_2_, which sustains H_2_DCF-DA oxidation throughout the acquisition period. These observations suggest that, although endogenous ROS are continuously regulated by antioxidant mechanisms, the presence of H_2_O_2_ at the tissue surface or within the tissue maintains an oxidative environment sufficient to sustain elevated ROS-associated fluorescence.

### 3.3 *In vivo* imaging of ROS dynamics in lettuce leaves following UV-C irradiation

Ultraviolet-C (UV-C) irradiation is a high-energy physical stress known to trigger endogenous ROS production in plant tissues, which may result in either cellular damage or beneficial physiological responses depending on the intensity and duration of the stress.

In this study, we applied three different UV-C doses (0.85, 1.71 and 3.42 kJ/m^2^) to evaluate their effects on ROS-associated fluorescence dynamics. The results indicated that UV-C exposure induces a measurable oxidative response compared to unstressed tissues at the early stage of acquisition (**Figure 4**). The unstressed leaves displayed the same pattern as the control of the H_2_O_2_-treated sample assay (**Figures 4A, B**; **Supplementary Figure S5A**), reflecting the basal oxidative activity of plant tissues mentioned above. Within UV-C-treated samples, fluorescence intensity increased with the applied dose, with the highest UV-C dose (3.42 kJ/m^2^) producing the strongest radiance values, followed by the intermediate (1.71 kJ/m^2^) and lowest (0.85 kJ/m^2^) doses. Statistical analysis presented in **Figure 4C** further revealed that the intermediate and high UV-C doses produced significantly higher fluorescence intensities than both the control and the low UV-C dose during the early stages of the experiment (0 to 60 min). These observations demonstrate the capacity of UV-C irradiation to trigger oxidative processes in plants and highlight the sensitivity of the *in vivo* fluorescence imaging approach for monitoring dynamic changes in plant redox status. However, unlike the progressive fluorescence increase observed following exogenous H_2_O_2_ application, UV-C exposure induced an immediate oxidative response followed by a gradual decline of fluorescence over time across all treatments. This kinetic profile suggests that UV-C irradiation triggers a rapid physiological response, likely before or at the onset of image acquisition. Such transient oxidative bursts following abiotic stress have been widely documented and are considered an early component of stress signaling (Apel and Hirt, 2004; Choudhury et al., 2017; Gill and Tuteja, 2010; Gómez et al., 2019). In addition, Rabelo et al. (2020) reported that UV-C irradiation stimulates mitochondrial activity and induces an early increase in ROS levels, potentially triggering downstream defense responses and changes in antioxidant metabolism. The progressive decline in ROS-associated fluorescence observed in our study may reflect a gradual shift in the balance between ROS production and scavenging as cellular regulatory mechanisms respond to UV-C-induced stress. Accordingly, differences between treatments became less pronounced over time and, by 120 min, fluorescence responses were not significantly different among treatments despite the dose-dependent responses observed at earlier time points. However, this interpretation remains hypothetical and should be further validated through complementary biochemical analyses, including the quantification of antioxidant enzyme activities and non-enzymatic antioxidants, in future studies.

**Figure 4:**
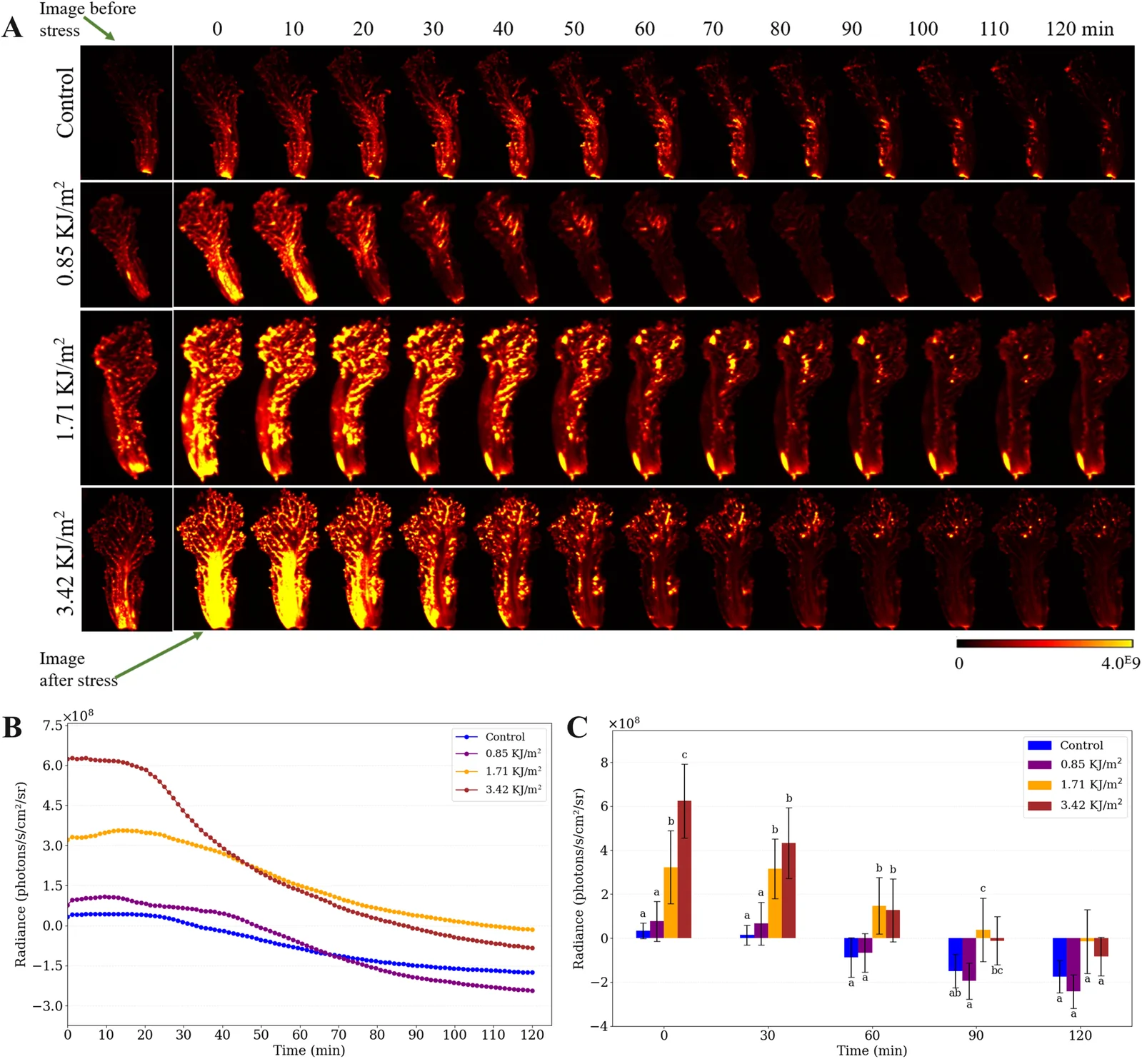
Temporal dynamics of UV-C-induced ROS accumulation in lettuce leaves. **(A)** Representative fluorescence images from a single biological replicate acquired before (control) and after UV-C irradiation at doses of 0.85, 1.71, and 3.42 kJ/m^2^. Fluorescence intensity reflects the accumulation of ROS detected by H_2_DCF-DA oxidation. The color scale represents radiance values (photons/sec/cm^2^/sr). **(B)** Time-resolved quantification of fluorescence intensity, expressed as mean radiance (photons/sec/cm^2^/sr) as a function of time using six biological replicates (n = 6). **(C)** Comparison of fluorescence intensity among treatments at selected time points (0, 30, 60, 90 and 120 min) based on six biological replicates (n = 6). Different letters indicate significant differences among treatments as determined by one-way ANOVA followed by Tukey’s multiple comparison test (p<0.05).

Furthermore, the results are consistent with previous studies identifying 0.85 kJ/m^2^ as a hormetic UV-C dose that preserves lettuce visual quality and induces resistance to *Botrytis cinerea*, whereas higher doses (1.71 and 3.42 kJ/m^2^) were associated with accelerated tissue deterioration after 5 and 4 days of storage, respectively (Attia et al., 2021; Ouhibi et al., 2014; Vàsquez et al., 2017). Hormetic doses are defined as treatments that stimulate plant defense mechanisms, thereby delaying senescence and contributing to the maintenance of membrane integrity (Acevedo Daza et al., 2024; Duarte-Serra et al., 2020). While these contrasting postharvest outcomes became evident after several days of storage, distinct ROS-associated fluorescence responses were detected within minutes following UV-C treatment. This temporal difference highlights the potential of the imaging approach for the rapid characterization of early oxidative stress responses that may influence subsequent quality changes during storage. The authors also reported no significant differences between the control and the 0.85 kJ/m^2^ treatment for oxidative stress indicators, including malonaldehyde (MDA), further supporting the classification of this dose as non-deleterious. Consistent with these findings, the fluorescence response induced by 0.85 kJ/m^2^ treatment in our study remained statistically indistinguishable from that of the control throughout the experiment, whereas 1.71 kJ/m^2^ and 3.42 kJ/m^2^ UV-C doses produced significantly higher fluorescence responses during early stages of acquisition (**Figure 4C**). This result was supported by the AUC analysis, which showed significantly greater cumulative fluorescence responses for the intermediate and high UV-C doses than the control and the low-dose treatment (**Supplementary Figure S5B**). These findings suggest that the *in vivo* fluorescence imaging approach is capable of discriminating between hormetic and damaging stressors, providing a rapid and non-destructive assessment of oxidative stress dynamics *in vivo* and in a time-resolved manner. Subsequently, *in vivo* imaging system-based fluorescence may serve as a valuable decision-support tool for optimizing postharvest treatment doses by enabling comparative evaluation of ROS-associated fluorescence responses under different stress intensities.

Although the present study demonstrates the potential of the *in vivo* fluorescence imaging approach for monitoring stress-induced changes in the oxidative status of whole lettuce leaves, limitations related to the plant material must be considered. All samples belonged to the same cultivar; however, they were obtained from a commercial supermarket, and therefore their developmental stage, preharvest environmental conditions, and postharvest handling conditions were unknown and not documented. This lack of control likely contributed to the variability observed among biological replicates. Such variability was particularly evident in the individual fluorescence profiles of biological replicates (**Supplementary Figures S4A**, **S5A**), where differences in fluorescence intensity were observed despite highly similar temporal response patterns within each treatment. Nevertheless, the consistent fluorescence kinetics observed across biological replicates indicate that the *in vivo* fluorescence imaging approach is sufficiently robust to detect treatment-dependent changes in oxidative status despite the inherent biological variability associated with commercially sourced plant material.

## 4 Conclusion

This study demonstrates the applicability of an *in vivo* fluorescence imaging system for the non-destructive, real-time monitoring of ROS-associated oxidative responses in whole postharvest plant tissues. By combining a whole-organ imaging platform with a ROS-sensitive fluorogenic probe (H_2_DCF-DA), the proposed workflow enables non-destructive and time-resolved assessment of oxidative responses at the tissue scale. Distinct fluorescence dynamics were observed depending on the applied stress, with exogenous H_2_O_2_ inducing a progressive increase in fluorescence consistent with sustained oxidant availability, whereas UV-C irradiation triggered a rapid and transient fluorescence response, characteristic of an early oxidative burst followed by a gradual decline. These stress-specific fluorescence patterns demonstrate the capability of the imaging approach to discriminate among different oxidative response behaviors in harvested lettuce leaves.

This approach provides a new experimental platform for investigating the temporal behavior of oxidative processes during postharvest storage and can be extended to other fruits and vegetables as well as to additional postharvest stressors, such as cold storage, mechanical damage, or pathogen exposure. Furthermore, the integration of complementary ROS-selective fluorescent probes together with biochemical analyses may further refine the interpretation of fluorescence signals by distinguishing the contribution of specific ROS species. Together, these advances could expand the application of *in vivo* fluorescence imaging in postharvest research, providing new insights into oxidative processes associated with storage, senescence, and quality deterioration, while supporting the development of improved postharvest management strategies.

## Data Availability

The raw data supporting the conclusions of this article will be made available by the authors, without undue reservation.
