# Supplementary material for: An in vivo fluorescence imaging approach for real-time monitoring of ROS-associated oxidative responses in harvested lettuce leaves
Source: Front Plant Sci. 2026 Oct 19;17:1923387. doi: 10.3389/fpls.2026.1923387 (PMC13724335; doi:10.3389/fpls.2026.1923387)
Supplement: Supplementary file 1 [file DataSheet1.docx]

**Supplementary Figures caption**

**Supplementary Figure S1:** Optimization of H_2_DCF-DA concentrations using 10, 30, 50, 70 and 100 µM in the presence of H_2_O_2_. (A) under tissue-free conditions (B) in lettuce leaves. Mean radiance (a), signal-to-background ratio (b), apparent kinetic constant k (c) and signal-to-noise ratio (SNR) derived from fluorescence intensity over time are used to assess probe sensitivity and reaction dynamics in the absence and presence of plant tissue.

**Supplementary Figure S2**: Image processing and fluorescence quantification pipeline.

**Supplementary Figure S3**: Effect of background image selection on fluorescence quantification following UV-C irradiation. Path (a) corresponds to background subtraction using the first image acquired after stress. Path (b) corresponds to background subtraction using an image acquired prior to stress, allowing selective quantification of stress-induced ROS-associated fluorescence.

**Supplementary Figure S4**: Fluorescence time-course profiles of individual biological replicates and quantitative fluorescence responses in lettuce leaves exposed to H_2_O_2_ treatment. Panel A shows fluorescence patterns of individual biological replicates (n=6) for the (a) control, (b) H_2_O_2_-treated lettuce leaves. Each curve represents a single lettuce leaf response. Panel B shows the area under the fluorescence-time curve (AUC) calculated for each biological replicate over the 120 min acquisition period and represented as mean ± SD. Statistical differences between treatments were evaluated using an unpaired student’s t-test. Asterisks indicate significant differences between treatments (***p<0.001, **p<0.01 and *p<0.05.

**Supplementary Figure S5**: Fluorescence time-course profiles of individual biological replicates and quantitative fluorescence responses in lettuce leaves exposed to UV-C treatments. Panel A shows fluorescence patterns of individual biological replicates (n=6) for the (a) control, (b) UV-C 0.85 KJ/m^2^, (c) UV-C 1.71 KJ/m^2^ and (d) UV-C 3.42 KJ/m^2^. Each curve represents a single lettuce leaf response. Panel B shows the area under the fluorescence-time curve (AUC) calculated for each biological replicate over the 120 min acquisition period and represented as mean ± SD. Different letters indicate significant differences among treatments as determined by one-way ANOVA followed by Tukey’s multiple comparison test (p<0.05).

Supplementary Figure S1: Optimization of H_2_DCF-DA concentrations using 10, 30, 50, 70 and 100 µM in the presence of H_2_O_2_. (A) under tissue-free conditions (B) in lettuce leaves. Mean radiance (a), signal-to-background ratio (b), apparent kinetic constant k (c) and signal-to-noise ratio (SNR) derived from fluorescence intensity over time are used to assess probe sensitivity and reaction dynamics in the absence and presence of plant tissue.

Supplementary Figure S2: Image processing and fluorescence quantification pipeline.

Supplementary Figure S3: Effect of background image selection on fluorescence quantification following UV-C irradiation. Path (a) corresponds to background subtraction using the first image acquired after stress. Path (b) corresponds to background subtraction using an image acquired prior to stress, allowing selective quantification of stress-induced ROS-associated fluorescence.

Supplementary Figure S4: Fluorescence time-course profiles of individual biological replicates and quantitative fluorescence responses in lettuce leaves exposed to H_2_O_2_ treatment. Panel A shows fluorescence patterns of individual biological replicates (n=6) for the (a) control, (b) H_2_O_2_-treated lettuce leaves. Each curve represents a single lettuce leaf response. Panel B shows the area under the fluorescence-time curve (AUC) calculated for each biological replicate over the 120 min acquisition period and represented as mean ± SD. Statistical differences between treatments were evaluated using an unpaired student’s t-test. Asterisks indicate significant differences between treatments (***p<0.001, **p<0.01 and *p<0.05.

Supplementary Figure S5: Fluorescence time-course profiles of individual biological replicates and quantitative fluorescence responses in lettuce leaves exposed to UV-C treatments. Panel A shows fluorescence patterns of individual biological replicates (n=6) for the (a) control, (b) UV-C 0.85 KJ/m^2^, (c) UV-C 1.71 KJ/m^2^ and (d) UV-C 3.42 KJ/m^2^. Each curve represents a single lettuce leaf response. Panel B shows the area under the fluorescence-time curve (AUC) calculated for each biological replicate over the 120 min acquisition period and represented as mean ± SD. Different letters indicate significant differences among treatments as determined by one-way ANOVA followed by Tukey’s multiple comparison test (p<0.05).
